# Early reduction of retinal thickness predicts physical and cognitive disability in newly diagnosed multiple sclerosis patients: results from a cross-sectional study

**DOI:** 10.1007/s10072-024-07664-9

**Published:** 2024-06-29

**Authors:** Simona Toscano, Clara Grazia Chisari, Alice Biondi, Francesco Patti

**Affiliations:** 1 Department “GF Ingrassia”, Section of Neurosciences, Neurology Clinic, University of Catania, 9126 Catania, Italy; 2 Department of Biomedical and Biotechnological Sciences (BIOMETEC), University of Catania, Catania, Italy; 3 Department “GF Ingrassia”, Section of Neurosciences, Multiple Sclerosis Center, Neurology Clinic, University of Catania, Via Santa Sofia 78, 95123 Catania, Italy

**Keywords:** Multiple sclerosis, Cognitive impairment, Optical coherence tomography, Retinal thickness, Physical disability

## Abstract

**Introduction:**

Retinal nerve fiber layer (RNFL) thickness is a promising biomarker of axonal loss and a potential outcome predictor in Multiple Sclerosis (MS). Cognitive impairment (CoI) exhibits a high prevalence in patients with MS (pwMS), even in the early phases of the disease. Our aim was to explore the role of RNFL thickness as a predictor of physical and cognitive disability in pwMS.

**Methods:**

All newly diagnosed pwMS referred to the MS centre of the University-Hospital “Policlinico-San Marco” between 2015–2019 were evaluated at baseline and at 3 years. RNFL and ganglion cell layer (GCL) thickness for right (r.e.) and left eyes (l.e.) were measured with Optical Coherence Tomography (OCT). Disability level and cognitive profile were assessed, using the Expanded Disability Status Scale (EDSS) and the Brief International Cognitive Assessment for Multiple Sclerosis (BICAMS) battery, respectively.

**Results:**

We consecutively enrolled 487 pwMS, including 68 (14.0%) with primary progressive MS (PPMS). At baseline, RNFL and GCL were bilaterally thinner in PPMS (r.e. 90.4 ± 12.7; l.e. 90.2 ± 13.5, and r.e. 80.1 ± 11.2; l.e. 80.3 ± 12.6, respectively) compared to relapsing–remitting MS (RRMS) (r.e. 94.6 ± 13.1; l.e. 94.3 ± 14.8, and r.e. 85.1 ± 9.5; l.e. 84.9 ± 9.3, respectively) (*p* < 0.01). Both groups exhibited reduced RNFL and GCL thickness, worse cognitive performance and higher EDSS scores at 3-years follow-up compared with baseline. RNFL thickness ≤ 88.0 μm was an independent predictor of CoI (OR = 5.32; 95% CI = 1.84–9.12; *p* = 0.02) and disability worsening (OR = 3.18; 95% CI = 1.21–10.33; *p* = 0.05).

**Discussion:**

RNFL thickness, as a biomarker of neurodegeneration, could be considered a predictive biomarker of cognitive degeneration and physical disability in MS.

## Introduction

Multiple Sclerosis (MS) is a chronic immune-mediated inflammatory disease of the central nervous system (CNS), characterized by both inflammation and neurodegeneration since its early phases. Over time, the accrual of axonal damage, consequent to demyelination, leads to the accumulation of physical disability and cognitive deterioration [1]. The assessment of the peripapillary retinal nerve fiber layer (RNFL) thickness with optical coherence tomography (OCT), a non-invasive instrument providing high-resolution tomographic sections of the retina, can be considered a reliable marker of axonal loss [2, 3]. RNFL is the innermost retinal layer, which is measured on a cross-sectional retinal image sampled along a 3.4-mm diameter circle centered on the optic nerve head [4].

Previous studies investigated the association between reduced RNFL and higher scores at the Expanded Disability Status Scale (EDSS), the most widely used clinical instrument to monitor disease severity and progression [5]. Whilst significant associations were found by some authors [6–8], other studies failed in demonstrating a correlation between RNFL and physical disability regardless of a previous history of optic neuritis (ON) or in detecting differences between relapsing and progressive MS phenotypes [9–13]. Beyond RNFL, which includes the unmyelinated axons of ganglion cell neurons, the measurement of the macular ganglion cell layer (GCL), alone or combined with the inner plexiform layers (GCIPL), is an even more accurate marker of neurodegeneration and correlates with brain atrophy [14–16].

Both RNFL and GCL thickness were investigated as indirect markers of CoI [7, 17]. It is well known that CoI exhibits a prevalence between 45–70% in patients with MS, higher in older and more severely disabled patients and in progressive phenotypes [18]. Further, CoI is associated with cortical atrophy at Magnetic Resonance Imaging (MRI) scans, which is itself a marker of neurodegeneration [19, 20]. Both RNFL thickness and GCL were found to be reduced in cognitively impaired patients and some studies found them to be reliable markers in predicting future CoI [7, 17, 21, 22].

In this perspective, we aimed to assess the role of RNFL and GCL in predicting CoI and physical disability over a 3-year follow-up in a population of patients newly diagnosed with MS without a history of ON.

## Methods

### Study population

We recruited all patients admitted to the MS Centre of Neurology Clinic at the University Hospital “Policlinico G. Rodolico” of Catania in the period between January 2015 and December 2019. All patients received a diagnosis of MS according to 2010 McDonald’s criteria [23] within 5 years from disease onset and were followed-up annually for at least 3 years. Only patients without a history of ON were finally included in the study population. All patients gave written consent to allow data collection and use for study purpose. We collected data about demographics, MS onset and course, EDSS, MRI and disease-modifying treatment (DMT) from a computerized database, iMed© (Merck Serono SA; Geneva), including real-time inserted data. Neurological examination and the attribution of EDSS scores were performed by experienced and certified neurologists for all patients according to clinical practice at baseline and then annually up to 3 years.

### Neuropsychological assessments

All enrolled subjects underwent a neuropsychological evaluation with the use of the Italian version of the Brief International Cognitive Assessment (BICAMS) [24, 25], a validated neuropsychological battery including the following tests:

1. Symbol Digit Modalities Test (SDMT), to provide a measure of speed in information processing. The total number of correct answers provided in 90 seconds is recorded and a threshold of 34.2 is commonly considered to pass the test [26];
2. California Verbal Learning Test-II (CVLT-II), which measures episodic verbal learning and memory by assessing encoding, recall and recognition in a single modality of item presentation (auditory-verbal). The test includes a 16-item word list, each belonging to one of four semantic categories, which is read aloud five times in the same order to the patient, asked to recall as many items as possible in any order [27];
3. Brief Visuospatial Memory Test Revised (BVMT-R), to evaluate visual learning. It consists of three learning trials for the patient, who is asked to reproduce on a sheet of paper six geometric figures previously shown for 10 seconds. Each drawing is evaluated according to accuracy and location and scored with 0–2 points, for a total score ranging from 0 to 12 for each trial. Delayed free recall of the same geometric figures is tested after 25 minutes (BVMT-R Delayed Recall) [28].

The presence of depressive symtoms was detected by administering the Beck Depression Inventory (BDI), a self-administered 21-items clinical interview investigating on the psychological and somatic symptoms of depression [29].

CoI was confirmed when failure of at least one neuropsychological test was recorded, identified as a score lower than 2 standard deviations (SD) from the normative values.

### Optical coherence tomography

OCT was performed at baseline and after 3 years (T2) with Stratus OCT (model Cyrrus 5000, Carl Zeiss Meditec, Dublin, CA). RNFL was acquired with the Optic Disc Cube 200 × 200 protocol that images the optic disc in a 6 mm × 6 mm region. The mean RNFL and values referred to individual quadrants were calculated. The annualized RNFL loss was considered as the difference between 3-year follow-up and baseline values calculated in the whole observation period divided by the number of years of observation.

Macular GCL was obtained using the Macular Cube 512 × 128 protocol that images a 6 mm × 6 mm area centered at the fovea. The GCL was calculated automatically over an elliptical annulus (2 mm × 2.4 mm radius), excluding the central foveal region (0.5 mm × 0.6 mm radius). Only well-focused and centred scans with a signal strength of at least 7 were included. Quality control and APOSTEL 2.0 recommendations according to published criteria were followed [30–32].

### Statistical analysis

Data were analyzed with STATA© (StataCorp, College Station, TX, United States, version 16.1). After assessing quantitative variables for normality with the Kolmogorov–Smirnov test, continuous variables were reported as mean and SD, or median and interval, as appropriate. Student’s t test and Mann–Whitney U Test (U) were used to compare continuous variables between groups. Categorical variables were expressed in frequencies and percentages, and compared between groups with Chi-square test (χ^2^).

A cut-off value of 88 μm was chosen as threshold for RNFL thickness, representing the lowest tertile of data distribution in our sample and a potential promising threshold according to a previous multicentre cohort study [33]. Cox proportional hazard models correcting for age, previous history of optic neuritis, disease duration and EDSS at baseline were used to test RNFL thickness ≤ 88 μm as a predictor of EDSS progression.

At the eye level, each outcome variable was examined using generalized estimating equation (GEE) models [34], which accounts for within-subject correlation. Univariate GEE models between each main variable (CoI and EDSS worsening) and covariate were carried out. We considered EDSS worsening as a change in EDSS by 1-point from a baseline score up to 5.5 or a 0.5-point increase from a baseline higher than 5.5 [32]. The considered covariates were: age, sex, educational level, age at onset, EDSS at diagnosis, disease duration, progressive phenotype, treatment with DMT, annualized RNFL loss, visual acuity (for both right and left eyes), RNFL and GCL thickness (for both right and left eyes), occurrence of relapses during the observation period, RNFL less than 88 µm.

Forward and backward methodologies were applied to construct a final multivariate model for each outcome with *p* < 0.2 at the univariate level being the criterion for being introduced (forward modeling) or retained (backward modeling) in the final multivariate model.

We tested all variables for collinearity by variance inflation factor (VIF) and excluded all variables from the regression analysis if VIF > 2.0 corresponding to an R^2^ of 0.60.A p value of < 0*.*05 was considered significant for all tests, which were 2-sided.

## Results

### Patients’ characteristics

We enrolled 487 patients diagnosed with MS, according to 2010 McDonald criteria [23], during the period between January 2015-December 2019. Of them, 419 (86.0%) exhibited a relapsing–remitting phenotype (RRMS) and 68 (14.0%) a primary progressive one (PPMS). Patients with PPMS (pwPPMS) were older than those with RRMS (pwRRMS) at onset (46.7 ± 11.7 vs 34.2 ± 11.7 years, *p* < 0.05) and at the time of diagnosis (52.7 ± 10.8 vs 37.0 ± 12.2 years, *p* < 0.001), and exhibited longer disease duration (107.4 ± 81.9 vs 66.3 ± 57.8; *p* < 0.05). Median EDSS at the time of diagnosis was significantly different between pwRRMS (2.0; 0.0–8.0) and pwPPMS (5.5; 1.5–8.0) (*p* < 0.001).

Clinical presentation at disease onset was different between groups, with pyramidal symptoms reported by 82.3% of PPMS and 59.4% of RRMS patients (*p* < 0.001), and visual symptoms described by 10.3% of PPMS and 24.1% of RRMS (*p* < 0.05). MRI characteristics of the study population are shown in Table 1. Among patients, 371 (88.5%) RRMS and 51 (75.0%) PPMS received DMT.

**Table 1:** MRI characteristics of the study population (487 patients) at baseline and at 3-year follow-up

	RRMS	PPMS	*p*
Baseline MRI			
Mean ± SD			
Brain			
T1	5.5 ± 7.7	9.5 ± 10	** < 0.01**
T2	22.3 ± 21.1	31.5 ± 29.2	** < 0.05**
Gd +	1 ± 2.6	0.3 ± 1.6	** < 0.01**
Spinal cord			
T1	0.03 ± 0.2	5.7 ± 14.1	** < 0.001**
T2	2.4 ± 2.1	6 ± 16.1	** < 0.05**
Gd +	0.6 ± 0.9	0.3 ± 0.4	** < 0.05**
3-year follow-up MRI			
Mean ± SD			
Brain			
T1	7.8 ± 8.6	13.7 ± 13.7	** < 0.05**
T2	25.1 ± 23.3	36.9 ± 33.9	** < 0.05**
Gd +	0.2 ± 1.7	0.1 ± 0.3	** < 0.05**
Spinal cord			
T1	0.02 ± 0.1	0	n.a
T2	2.4 ± 2.5	3.9 ± 2.3	** < 0.05**
Gd +	0.07 ± 0.3	0.1 ± 0.2	** < 0.01**

*MRI* Magnetic Resonance Imaging, *Gd* + gadolinium-enhanced, *SD* standard deviation, *n.a.* not applicable

### Baseline and follow-up neurophthalmological evaluations

**Fig. 1:**
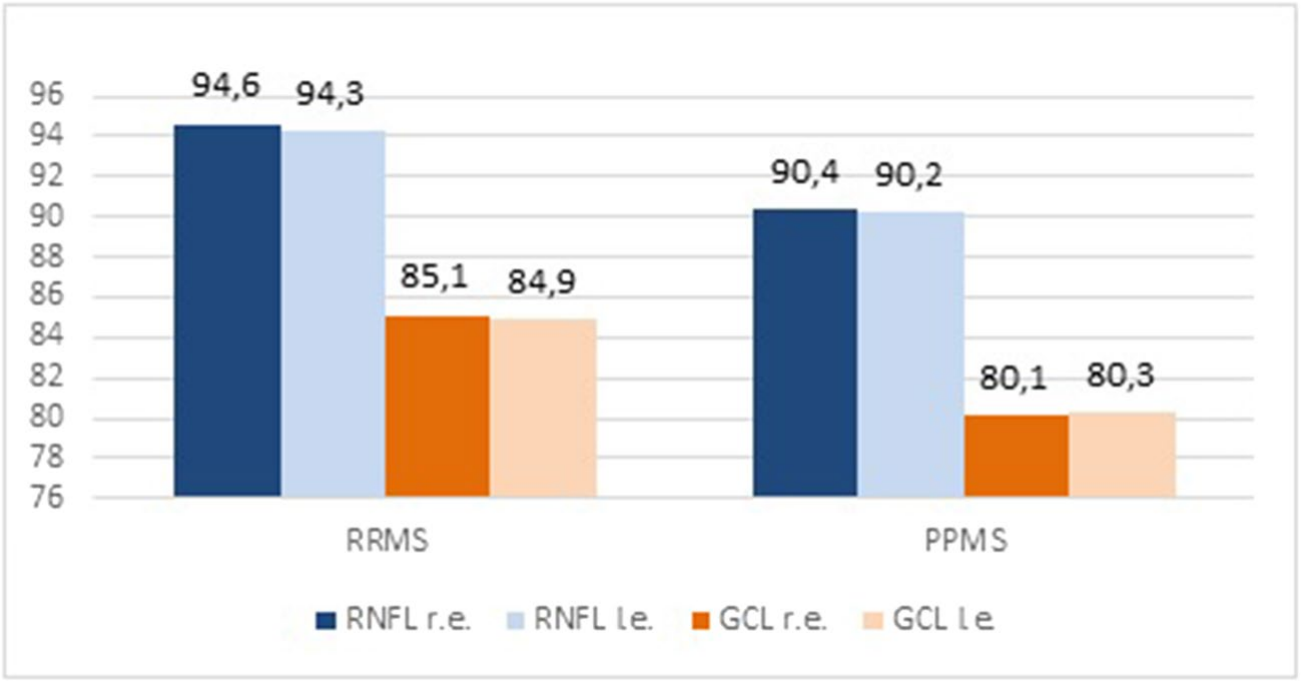
OCT parameters detected at baseline in RRMS and PPMS patients. RRMS: relapsing-remitting multiple sclerosis; PPMS: primary progressive multiple sclerosis; RNFL: retinal nerve fiber layer; GCL: ganglion cell layer; r.e.: right eye; l.e.: left eye

**Fig. 2:**
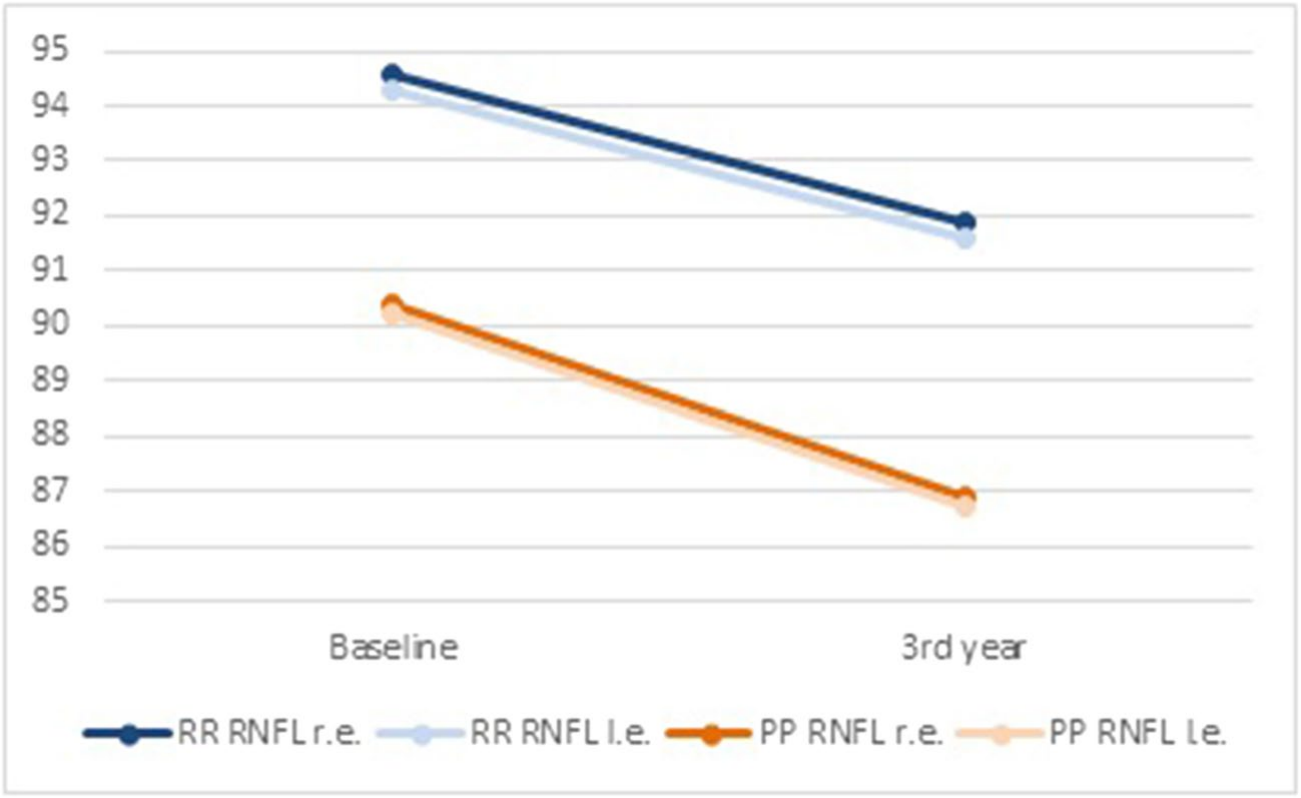
RNFL thickness at baseline and at 3-year follow-up in RRMS and PPMS patients. RRMS: relapsing-remitting multiple sclerosis; PPMS: primary progressive multiple sclerosis; RNFL: retinal nerve fiber layer; r.e.: right eye; l.e.: left eye

**Fig. 3:**
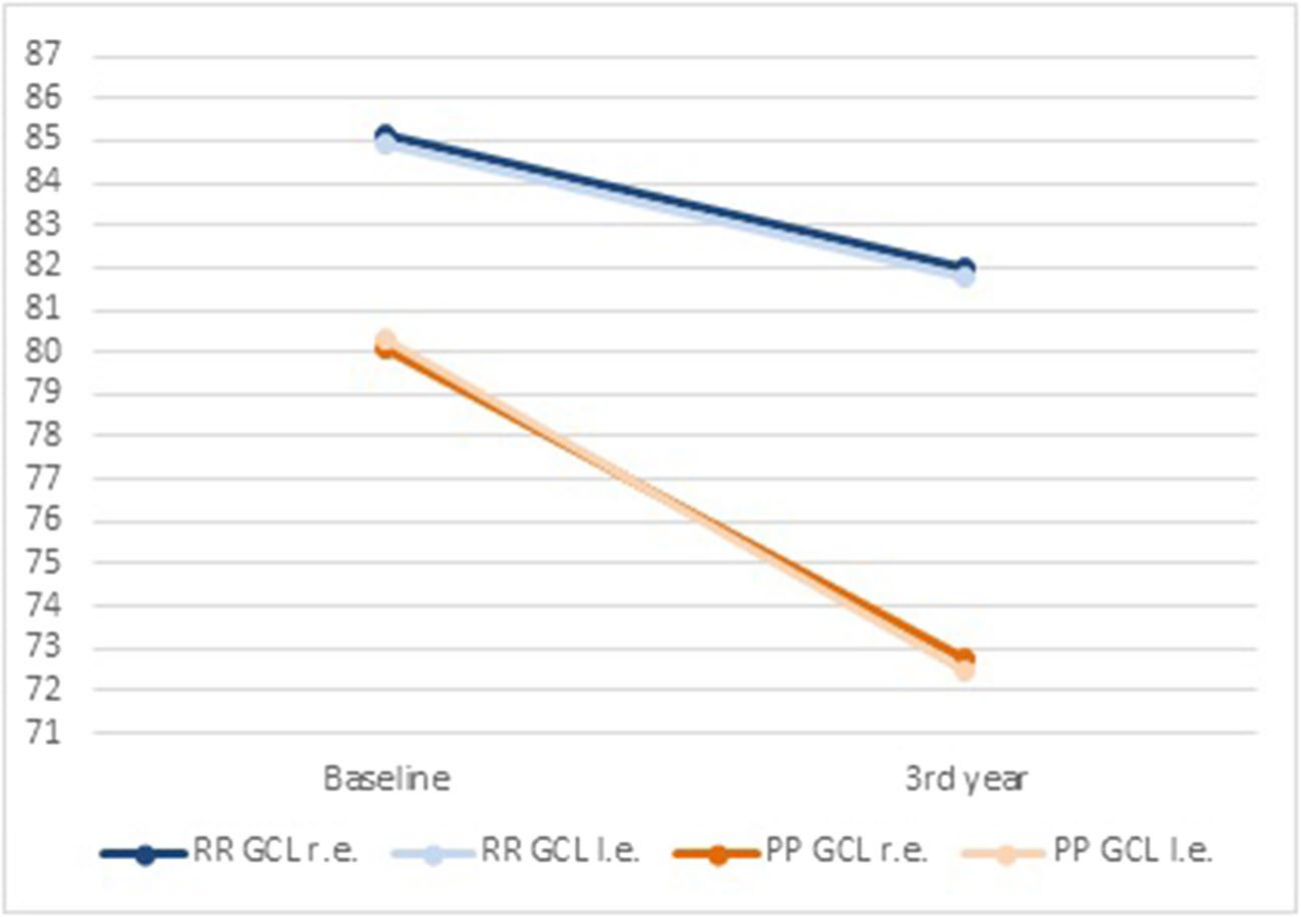
GCL thickness at baseline and at 3-year follow-up in RRMS and PPMS patients. RRMS: relapsing-remitting multiple sclerosis; PPMS: primary progressive multiple sclerosis; GCL: ganglion cell layer; r.e.: right eye; l.e.: left eye

At baseline, pwPPMS exhibited thinner RNFL (r.e., 90.4 ± 12.7; l.e. 90.2 ± 13.5) compared with pwRRMS (r.e. 94.6 ± 13.1; l.e. 94.3 ± 14.8) (*p* < 0.01). Similarly, reduced GCL thickness was detected in PPMS (r.e., 80.1 ± 11.2; l.e. 80.3 ± 12.6) compared with RRMS (r.e., 85.1 ± 9.5; l.e. 84.9 ± 9.3) (*p* < 0.05) (Table 2; Fig. 1). RNFL thickness decreased bilaterally in both pwRRMS and pwPPMS at 3-year follow-up (*p* < 0.01) (Table 3; Fig. 2). Similarly, a thinner GCL was detected in both eyes when OCT was performed at follow-up compared with baseline values in both pwRRMS (*p* < 0.01) and pwPPMS (*p* < 0.001) (Table 3; Fig. 3). At baseline, no differences were observed in terms of visual acuity in patients with RRMS and PPMS (Table 2). After 3-years follow-up, visual acuity did not significantly modify in both RRMS and PPMS groups (Table 3).

**Table 2:** OCT parameters, visual acuity and neuropsychological assessments performed at baseline in RRMS and PPMS patients

	RRMS (419)	PPMS (68)	*p*
VA r.e., LogMAR			
Mean ± SD	0.06 ± 0.02	0.07 ± 0.05	**n.s**
VA l.e., LogMAR			
Mean ± SD	0.06 ± 0.03	0.07 ± 0.05	**n.s**
RNFL r.e., µ			
Mean ± SD	94.6 ± 13.1	90.4 ± 12.7	** < 0.01**
RNFL l.e., µ			
Mean ± SD	94.3 ± 14.8	90.2 ± 13.5	** < 0.01**
GCL r.e., µ			
Mean ± SD	85.1 ± 9.5	80.1 ± 11.2	** < 0.05**
GCL l.e., µ			
Mean ± SD	84.9 ± 9.3	80.3 ± 12.6	** < 0.05**
SDMT			
Mean ± SD	36.1 ± 12.2	24.9 ± 7.7	** < 0.01**
CVLT tot			
Mean ± SD	9.9 ± 3.1	7.3 ± 3.9	** < 0.01**
BVMT I			
Mean ± SD	9.0 ± 3.0	8.0 ± 2.9	n.s
BVMT II			
Mean ± SD	9.8 ± 2.6	8.0 ± 3.0	** < 0.01**
BVMT III			
Mean ± SD	10.7 ± 2.9	9.0 ± 4.1	** < 0.01**
CoI			
N (%)	152 (36.3)	31 (45.7)	** < 0.05**

*VA* visual acuity, *LogMAR* logarithm of minimal angle of resolution, *OCT* optical coherence tomography, *RRMS* relapsing–remitting multiple sclerosis, *PPMS* primary progressive multiple sclerosis, *RNFL* retinal nerve fiber layer, *GCL* ganglion cell layer, *r.e.* right eye, *l.e.* left eye, *µ* micron, *9HPT* 9-hole peg test, *d* dominant, *nd* non-dominant, *sec* seconds, *T25FWT* timed 25-foot walk test, *SDMT* symbol digit modalities test, *CVLT* California verbal learning test, *BVMT* brief visuospatial memory test, *CoI* cognitive impairment, *SD* standard deviation

**Table 3:** Results from OCT assessment at baseline in the study population (487 patients)

	RRMS			PPMS		
	Baseline	Follow-up	*p*	Baseline	Follow-up	*p*
VA r.e., LogMAR						
Mean ± SD	0.06 ± 0.02	0.07 ± 0.04	**n.s**	0.07 ± 0.05	0.08 ± 0.04	**n.s**
VA l.e., LogMAR						
Mean ± SD	0.06 ± 0.03	0.07 ± 0.05	**n.s**	0.07 ± 0.05	0.07 ± 0.06	**n.s**
RNFL r.e., µ						
Mean ± SD	94.6 ± 13.1	91.9 ± 14.6	** < 0.01**	90.4 ± 12.7	86.9 ± 16.9	** < 0.01**
RNFL l.e., µ						
Mean ± SD	94.3 ± 14.8	91.6 ± 16.0	** < 0.01**	90.2 ± 13.5	86.7 ± 15.3	** < 0.01**
GCL r.e., µ						
Mean ± SD	85.1 ± 9.5	82.0 ± 11.1	** < 0.01**	80.1 ± 11.2	72.8 ± 13.8	** < 0.001**
GCL l.e., µ						
Mean ± SD	84.9 ± 9.3	81.8 ± 11.9	** < 0.01**	80.3 ± 12.6	72.5 ± 15.2	** < 0.001**

*RRMS* relapsing–remitting multiple sclerosis, *PPMS* primary progressive multiple sclerosis, *RNFL* retinal nerve fiber layer, *GCL* ganglion cell layer, *r.e.* right eye, *l.e.* left eye, *µ* micron, *SD* standard deviation

### Baseline and follow-up NPS evaluation

Comprehensively, 152 of 419 RRMS (36.3%) and 31 of 68 PPMS (45.6%) subjects were cognitively impaired at baseline evaluation (Table 2). PwRRMS exhibited significantly lower scores at 3-year SDMT (33.8 ± 16.6) compared with baseline values (36.1 ± 12.2; *p* < 0.05), while no differences were detected in other tests (Table 4).

**Table 4:** Neuropsychological examination at baseline and at 3-year follow-up in the study population (487 patients)

	RRMS			PPMS		
	Baseline	Follow-up	*p*	Baseline	Follow-up	*p*
SDMT	36.1 ± 12.2	33.8 ± 16.6	** < 0.05**	24.9 ± 7.7	19.8 ± 8.2	** < 0.001**
CVLT	9.9 ± 3.1	9.4 ± 2.9	n.s	7.3 ± 3.9	7.1 ± 3.1	** < 0.01**
BVMT I	9.0 ± 3.0	8.8 ± 3.7	n.s	8.0 ± 2.9	6.4 ± 3.5	** < 0.01**
BVMT II	9.8 ± 2.6	9.3 ± 2.7	n.s	8.0 ± 3.0	6.5 ± 2.1	** < 0.01**
BVMT III	10.7 ± 2.9	9.6 ± 4.3	n.s	9.0 ± 4.1	6.9 ± 4.2	** < 0.01**
CoI	152 (36.3)	184 (43.9)	** < 0.05**	31 (45.7)	37 (54.4)	** < 0.05**

*RRMS* relapsing–remitting multiple sclerosis, *PPMS* primary progressive multiple sclerosis, *SDMT* symbol digit modalities test, *CVLT* California verbal learning test, *BVMT* brief visuospatial memory test, *CoI* cognitive impairment, *SD* standard deviation

**Fig. 4:**
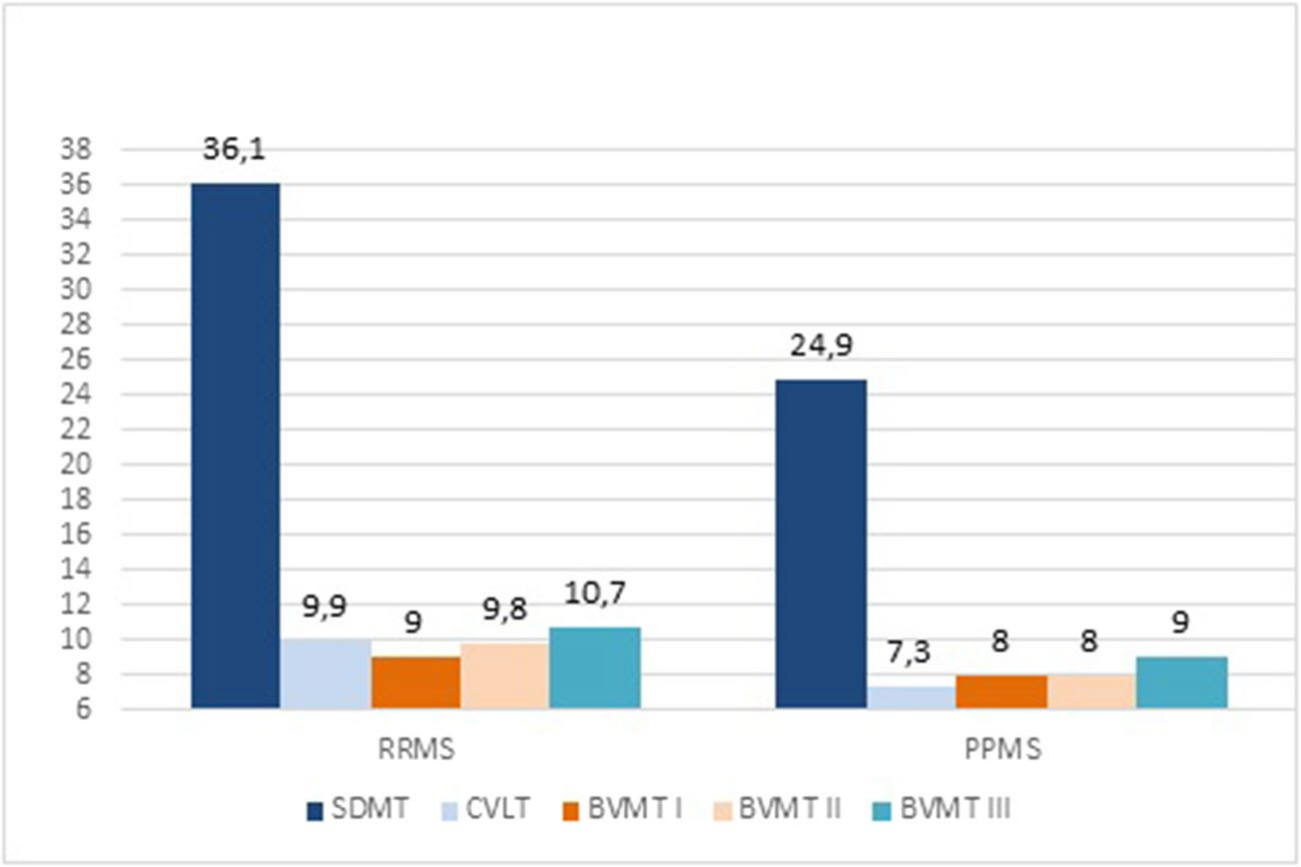
Cognitive performance of RRMS and PPMS patients at baseline and at 3-year follow-up at different neuropsychological tests (average scores). RRMS: relapsing–remitting multiple sclerosis; PPMS: primary progressive multiple sclerosis; SDMT: symbol digit modalities test; CVLT: California verbal learning test; BVMT: brief visuospatial memory test

PwPPMS performed worse than pwRRMS at baseline SDMT (respectively, 24.9 ± 7.7 vs 36.1 ± 12.2; *p* < 0.01), CVLT (7.3 ± 3.9 vs 9.9 ± 3.1; *p* < 0.01), BVMT II (8.0 ± 3.0 vs 9.8 ± 2.6; *p* < 0.01) and BVMT III (9.0 ± 4.1 vs 10.7 ± 2.9; *p* < 0.01) (Fig. 4). Further, they exhibited a significant worsening in cognitive performance at all tests at 3-year follow-up (*p* < 0.01) (Table 4). The prevalence of CoI at 3-year follow-up was significantly higher than at baseline for both RRMS (respectively, 43.9% vs 36.3%; *p* < 0.05) and PPMS patients (respectively, 54.4% vs 45.7%; *p* < 0.05).

### Predictors of cognitive impairment and EDSS worsening

Results from the GEE analysis are reported in Table 5. Patients with RNFL thickness less or equal to 88 µm at baseline exhibited a significantly increased odds of being cognitively impaired after 3 years from diagnosis. Progressive phenotype, RNFL ≤ 88 µm confirmed their role as independent risk factors for the detection of CoI at 3-year follow-up, along with higher disease duration and annualized RNFL loss. Conversely, a higher educational level was a protective factor for the outcome after 3 years from diagnosis.

**Table 5:** Results from the GEE analysis exploring the risk of developing Cognitive Impairment and EDSS worsening at 3-year follow-up in the study population

	CoI	EDSS worsening
Age	-0.61 (-0.41, 1.52)	0.98 (0.45, 2.25)
Sex	-0.54 (-0.35, 1.01)	0.84 (0.12, 3.51)
Educational level	-1.68 (-0.15, -3.19)*	-1.04 (-0.65, 1.51)
Annualized RNFL loss		
RE	2.08 (1.09, 4.79)**	1.63 (1.26, 4.07)**
LE	2.09 (1.25, 4.82)**	1.62 (1.24, 4.12)**
RNFL (µm)		
RE	1.18 (0.93, 5.81)	0.23 (0.06, 0.47)
LE	1.19 (0.95, 5.82)	0.22 (0.08, 0.45)
RNFL < 88 µm (yes/no)		
RE	1.64 (1.19, 5.79)*	1.43 (1.06, 5.47)
LE	1.69 (1.21, 5.82)*	1.42 (1.08, 5.45)
GCL (µm)		
RE	1.22 (0.91, 5.25)	2.09 (0.87, 8.21)
LE	1.27 (0.91, 4.59)	1.85 (0.25, 7.15)
Age at onset	0.58 (-2.31, 3.09)	0.87 (-0.25, 1.98)
EDSS at diagnosis	1.31 (-0.92, 4.29)	2.51 (1.35, 6.08)**
Disease duration	3.39 (1.36, 5.15)**	2.32 (1.01, 7.21)**
Treatment (yes/no)	-0.31 (-1.06, 4.36)	-0.25 (-1.09, -0.96)
Progressive phenotype	1.31 (0.95, 5.69)*	2.05 (1.09, 6.26)*
Visual Acuity		
RE	1.08 (0.88, 4.19)	1.03 (0.86, 3.77)
LE	1.09 (0.85, 4.28)	1.02 (0.81, 3.84)
Relapse during the observation period (yes/no)	1.48 (-0.12, 3.55)	1.62 (-0.93, 2.02)

Each cell contains the beta coefficient (95% confidence interval) for the outcome in that row as a function of the covariate in that column adjusted for all other covariates and within subject correlation using GEE models

*RNFL* retinal nerve fiber layer, *RE* right eye, *LE* left eye; *: *p* < 0.05; **: *p* < 0.01

EDSS worsening at 3 years was independently predicted by EDSS at diagnosis, disease duration, progressive course. Additionally, a higher annualized RNFL loss was associated to increased risk of EDSS worsening at 3-year follow-up, while the chosen cut-off of 88 µm was not a predictor for EDSS worsening in the observation period.

## Discussion

Results from our study supported the reliability of RNFL and GCL as potential predictive biomarkers for the development of physical and cognitive deterioration during follow-up in pwMS. Both pwRRMS and pwPPMS exhibited significantly lower values of RNFL and GCL thickness, higher EDSS scores and worse cognitive performance over a 3-year follow-up compared with baseline, although the latter performed worse at all assessments already at baseline.

Several studies reported a significant difference in RNFL and GCL thickness in pwMS compared with controls, regardless of a previous history of ON [7, 13, 14, 35, 36]. Particularly, the measurement of GCL proved to be even more sensitive in the early phases of the disease and strongly associated with brain atrophy [14–16]. Further, several studies reported evidence of RNFL thickness reduction over time [15, 37–39], including a recent 2-year prospective study involving 135 pwMS and 16 controls [15]. Particularly, a significant inner retinal layer thinning was reported in pwMS compared with healthy controls in a 3-year prospective multicenter study, as well as a greater brain volume decrease [36]. However, when comparing these parameters among MS phenotypes, results were controversial.

In a study involving 326 pwMS and 94 controls, progressive MS patients (PMS) were characterized by decreased RNFL thickness compared with RRMS, and both groups exhibited a smaller thickness compared with controls, regardless of previous ON [13]. Another study found a significant thinning of RNFL, GCIPL and outer plexiform layer in PMS compared to RRMS [8]. Differently, no distinction emerged between pwPPMS and pwRRMS without ON in some studies [9, 35], while others detected differences in RNFL only or particularly in SPMS rather than PPMS compared with controls [14, 40]. Still, a few studies compared only progressive phenotypes with controls [40] or considered them as a single group, without distinction between SPMS and PPMS [35].

As expected according to the natural disease course, in our study pwPPMS were older and more physically and cognitively impaired at the time of diagnosis, compared with pwRRMS. Therefore, it was not unforeseeable that pwPPMS exhibited greater disability and worse cognitive performances at 3-year follow-up. It is interesting that RNFL and GCL thickness reflected this trend, with a more pronounced reduction in pwPPMS both at baseline and at follow-up.

We used a threshold of 88.0 µm, which corresponded to the lowest tertile of data distribution in our sample, to define a significant reduction in RNFL thickness. PwMS with RNFL thickness lower than the chosen cut-off exhibited an increased risk to develop CoI in our population. This is in line with results from a cross-sectional study involving 217 pwMS and evaluating possible associations between inner retinal layer atrophy and CoI [21]. Not only cognitively impaired patients exhibited significantly lower mean RNFL and GCIPL than cognitively preserved ones, but RNFL lower than 85.0 µm and GCIPL below 88.1 µm, which were the median values in the data distribution, were respectively associated to fourfold and threefold increased odds of subsequent CoI [21]. As in our study, SDMT was used to assess the presence of CoI. It was the only test in our study population, within BICAMS battery, which was sensitive to cognitive worsening of both pwRRMS and pwPPMS over time, thus confirming its clinical usability as a first-line screening test for CoI [41]. Additionally, previous results indicated a good correlation between visual test performance and processing speed, more than memory function [42]. As expected, pwPPMS achieved lower scores at all cognitive tests and exhibited a significantly higher prevalence of CoI both at baseline (45.7%) and at follow-up (54.3%) compared with RRMS (36.3% and 43.8%, respectively). Indeed, as reported by previous studies [43], EDSS at diagnosis and progressive phenotype were known independent risk factors for the detection of CoI, both at baseline and follow-up, while a higher educational level was a protective factor for the investigated outcome. Other studies reported the association between CoI and the detection of smaller RNFL and GCIPL thickness [7, 17, 22, 44, 45]. A large multicenter prospective one reported a significant association between worse cognitive performance and a thinner RNFL at baseline [45]. Particularly, patients with RNFL thickness values in the lowest quintile were 11% more likely to fail at least one neuropsychological test and those in the two thinnest quintiles exhibited a double risk of perfoming worse at follow-up cognitive assessments.

In our study, the annualized RNFL loss in pwMS without ON was an independent predictor of disability worsening, regardless of treatment with DMT. As expected, EDSS at diagnosis, disease duration and progressive course were also predicting factors for EDSS worsening at follow-up. In another study, a reduced RNFL thickness was associated with a worse cognitive performance at SDMT, as well as with higher physical disability, confirmed by higher EDSS scores [46].

In a 3-year prospective study involving 141 RRMS patients, an annual RNFL thinning rate higher than 1.5 µm distinguished between stable and progressing patients with a sensitivity of 76.1% and a specificity of 90.0%, and such a threshold was associated with a 15-fold increased risk of clinically progressing MS [47]. Faster rates of annualized GCL thinning were associated with clinical and radiological disease-activity and disability progression during follow-up [48] and the annualized rates of thinning of RNFL and GCIPL were greater in pwMS who converted to SPMS than in those who did not convert, particularly before conversion [49]. Additionally, a multicentre cohort study found an association between a RNFL below 88 µm and a double risk of disability worsening during a 3-year follow-up [33].

Comprehensively, our results supported the use of RNFL and GCL as a biomarker of axonal damage since the early phases of the disease and, more notably, in the absence of previous ON. A threshold of 88 μm, further, could be helpful to distinguish pwMS at high-risk of developing cognitive disability over a short-term follow-up and higher rates of annualized RNFL loss predict increased risk of EDSS worsening. This further supports preliminary evidence about the association between RNFL thinning and progression independent from relapse activity (PIRA) [47], which accumulates since the very early phases of MS course in all phenotypes. Indeed, despite pwPPMS exhibited worse physical and cognitive performances than pwRRMS at all time-points in our study, RNFL and GCL thinning significantly predicted the development of physical and cognitive disability over years in both groups of patients, corroborating the recently proposed “one-MS hypothesis”, characterized by a unique underlying smouldering process, reflected by RNFL and GCL thinning, and by a superimposed focal inflammatory activity which differs among clinical phenotypes.

Our study has several limitations. First, we did not investigate the presence of cortical atrophy or the location of demyelinating lesions in specific brain regions, which are known to be relevant for cognitive functions [20]. Assessing the association with radiological characteristics could have further enhanced the reliability of OCT in predicting CoI and disability worsening, with the advantage of providing easier, shorter, and less expensive evaluations compared with MRI.

Still, we did not deepen the impact of different DMT on OCT parameters over time, as well as on cognitive performance and physical disability. Further, patients diagnosed with SPMS were not present in our study population since newly diagnosed patients were enrolled. However, this could be considered an advantage in order to strictly compare pwPPMS and pwRRMS. Indeed, progressive patients have often been considered as a single group in some studies exploring the predictive role of OCT, despite some authors reported differences in RNFL thickness between SPMS and PPMS [14, 35, 40]. Additionally, we disposed of a large amount of data from 487 MS patients with a recent diagnosis and naïve to any DMT, analyzed at two different timepoints.

In this view, we believe that the use of OCT, already implemented in the diagnostic work up, should definitely be considered as a valuable resource to monitor disease course in pwMS, providing relevant information by performing a rapid, non-invasive and quantitative evaluation.

## Data Availability

The datasets analyzed during the current study are available from the corresponding author on reasonable request.
